# Impact of the Price of Gifts From Patients on Physicians’ Service Quality in Online Consultations: Empirical Study Based on Social Exchange Theory

**DOI:** 10.2196/15685

**Published:** 2020-05-05

**Authors:** Yanan Wang, Hong Wu, Chenxi Xia, Naiji Lu

**Affiliations:** 1 Huazhong University of Science and Technology Wuhan China; 2 Jiangxi Engineering Laboratory on Radioactive Geoscience and Big Data Technology East China University of Technology Nanchang, Jiangxi China

**Keywords:** gift giving, gift price, service price, online consultation service quality, information support, emotional support, online health communities

## Abstract

**Background:**

Gift giving from patients to physicians, which is prohibited in traditional clinical settings in China, has been found to occur in online health communities. However, there is debate on the validity of online gifts since physicians gain an economic benefit. Moreover, the potential impact of these gifts, particularly with respect to the financial value of the gift, on the online consultation service quality remains unexplored.

**Objective:**

The aim of this study was to explore the impact of gift price on the quality of physicians’ online consultation service. Insight into this impact is expected to help resolve existing debate on the appropriateness of the gift-giving practice in online consultations.

**Methods:**

A dataset of 141 physicians and 4249 physician-patient interactions was collected from the Good Physician Online website, which is the largest online consultation platform in China. Based on social exchange theory, we investigated how gift price affects the quality of physicians’ online consultation service and how this impact changes according to the physician’s service price and number of all gifts received. Manual annotation was used to identify the information support paragraphs and emotional support paragraphs in the answers of physicians. The quality of the information support paragraphs, rather than the complete answer, was used to test the robustness of our model.

**Results:**

Gift price had a positive impact on the quality of physicians’ online consultation service (β=4.941, *P*<.01). This impact was negatively mediated by both the physician’s service price (β=–9.245, *P*<.001) and the total number of gifts they received (β=–5.080, *P*<.001).

**Conclusions:**

Gift price has a positive impact on physicians’ online behavior, although the impact varies among physicians.

## Introduction

### Background

Gift exchange, including giving, receiving, and reciprocating, is generally defined as the circulation of goods to promote ties and bonds between individuals [1]. Generally, gift giving has benefits for emotional expression and relationship building in interpersonal communication [2]. However, gifts exchanged between physicians and patients have been viewed as a form of illegal and unethical payment [3], which can potentially weaken the trust and deteriorate these relationships [4]. Hence, it has been forbidden for physicians to receive gifts from patients in China [4].

However, the practice of online gift giving has emerged with the development of online health communities. Online gifts were initially launched to provide patients with a channel to express gratitude to physicians, and debates about the validity of gifts in online health communities have sparked given the potential for bringing financial benefits to physicians. Some patients consider that online gift giving in online health communities represents a form of extortion with a bribe to a certain degree [5,6], whereas users of some online platforms regard it as a form of respect by acknowledging the knowledge labor of physicians [7]. In addition, physicians express different attitudes with respect to receiving online gifts. Approximately 70% of physicians stated that online gift giving is reasonable, 25% stated that it may make people uncomfortable, and 3% disapproved of this practice overall [7]. The root of these debates is the possible impact of the financial value of the gift on the quality of physicians’ online consultation service, which remains unclear.

In addition to these debates, online gifts have also attracted the attention of some scholars. Zhao et al [8] studied physician-patient interactions on the online health community Good Physician Online and confirmed that online gifts from patients could improve physicians’ online response rate. However, this study did not delve into the possible effects of the gift price. Gift giving, as a form of social exchange [9], follows the rules of social exchange theory (SET). Therefore, the aim of this study was to use SET to empirically explore how patients’ gift-giving behaviors affect physicians’ online consultation service quality from the perspective of gift price, which could help to resolve existing debates.

The deprivation-satisfaction proposition [10] in SET, which is based on the marginal diminishing effect [11] in economics, holds that the more people that receive a certain reward, the lower the perceived value of subsequent or similar rewards will be. Therefore, we considered that the total number of gifts a physician receives may have a negative moderating impact on the relationship between gift price and the physicians’ online consultation service quality. Finally, comparison level theory, also included in SET, holds that individuals will consider an acceptable outcome at a comparative level according to their previous experiences, which could affect their perception and judgment of subsequent outcomes. Similarly, the physicians’ service price might be considered the comparison level, which could affect their perception of the gift price. Thereby, we surmised that a physician’s service price might also have a negative moderating effect on the impact of gift price on the quality of their online consultation service. More specifically, this study addressed the following three research questions: (1) how does gift price affect the quality of physicians’ online consultation service? (2) how does the impact of gift price on the quality of physicians’ online consultation service differ for physicians with different service prices? and (3) how does the impact of gift price on the quality of physicians’ online consultation service differ for physicians receiving different numbers of online gifts?

### Related Research

SET is one of the most influential conceptual paradigms for understanding workplace behavior [12]. This theory can be traced back to at least the 1920s [13,14], bridging such disciplines as anthropology [15,16], social psychology [10,17,18], and sociology [19]. Although different views of social exchange have emerged, it is widely accepted that social theory involves a series of interactions that generate obligations [20], and these interactions are usually perceived as interdependent and contingent on the actions of another person [19].

Along with rapid development of the internet, online communities have become popular platforms for carrying out routine activities of daily life. Online interaction is an important part of the online community. Such interactions represent social exchanges between participants, which occur in a network context [21]. Therefore, many human behaviors on online communities have been studied in the context of SET. For example, some scholars have focused on purchase intention [22,23], online trust [24,25], and the information contribution intention of consumers in the marketing field [26], along with aspects of self-disclosure [27], reciprocal intention of knowledge [28], and member commitment [29].

Social exchange behaviors in online health communities mainly occur between physicians and patients. Researchers have traditionally explained user behaviors in these communities based on SET, such as the knowledge sharing intention [30], influence factors of providing social support [31], and the motivation of physicians to participate in online health communities [32]. Moreover, gift giving is a form of social exchange, and some researchers have also studied the influencing factors of gift-giving frequency in social network services based on SET [33]. However, less attention has been paid to gift-giving behaviors in online health communities. Therefore, the aim of the present study was to explore gift-giving behaviors in online health communities based on SET.

### Hypotheses Development and Research Model

#### Gift Price and Quality of Physicians’ Online Consultation Service

Reciprocity, an important prerequisite for continuous exchange, refers to the fact that one has an obligation to return another’s favor [17,34]. Moreover, reciprocity is not only the basis of gift exchange but is also the impetus for the flow of gifts [35]. In addition, the law of equality sets norms for social exchange according to the proportion of pay and return in the process [19]. Generally, interpersonal relationships can only be maintained based on the principles of reciprocity and equality [10].

Individuals are generally motivated to interact with others when they expect positive results [36]. Likewise, patients’ gift-giving behaviors in online health communities reflect their expectations for positive results such as higher quality of consultation services and more harmonious physician-patient relationships. However, a physician’s participation in an online health community is a social exchange process of professional capital for social and economic returns [32]. Therefore, a social exchange process could form through patients giving gifts and physicians reciprocating. According to the principles of reciprocity and equality, a physician may provide an online consultation service with relatively higher quality according to the price of the gift that they received. Therefore, we proposed hypothesis 1: gift price has a positive impact on the quality of physicians’ online consultation services.

#### Moderating Role of Gift Number

The marginal diminishing effect principle [37] posits that the value of a commodity depends on the perceived utility value by people. From this theory, the value of the marginal units of a commodity will decrease with an increase in the number of this commodity. Moreover, Homans’ [10] deprivation-satisfaction proposition, according to the marginal diminishing effect, holds that the more people that receive a certain reward, the lower the perceived value of subsequent or similar rewards will be.

The reason for this marginal diminishing effect is that from the perspectives of physiology and psychology, both satisfaction and stimulation will decline with each newly added consumption unit [38]. Similarly, early gifts can bring more pleasure and stimulus to physicians. However, as the number of gifts gradually increases, physicians may regard the gift-giving behaviors of patients as a normal part of their interactions. Thus, we hypothesized that physicians’ perceived value of gifts would gradually decline as the number of gifts they receive increases. Thus, hypothesis 2 is that the number of gifts that physicians receive negatively moderates the impact of gift price on the quality of physicians’ online consultation service.

#### Moderating Role of Physicians’ Service Price

Comparison level theory [18] was initially developed to study people’s assessments of interpersonal relationships, indicating that people will assess a relationship by comparing it with a known comparison level, which could be determined from one’s previous relationships or similar relationships [18]. Specifically, whether a relationship is attractive or satisfactory can be determined by comparing it with the comparison level.

In online consultations, physicians may first receive a consulting fee and thus form a comparison level. When they receive a gift during this process, they may compare the price of the gift with the comparison level. Therefore, a physician’s satisfaction with the gift price would be lower when their service price is higher. Furthermore, the positive impact of gift price on the quality of the physician’s online consultation service will be weaker when their service price is higher, and vice versa, leading to hypothesis 3: service price negatively moderates the relationship between gift price and the quality of physicians’ online consultation services.

**Figure 1:**
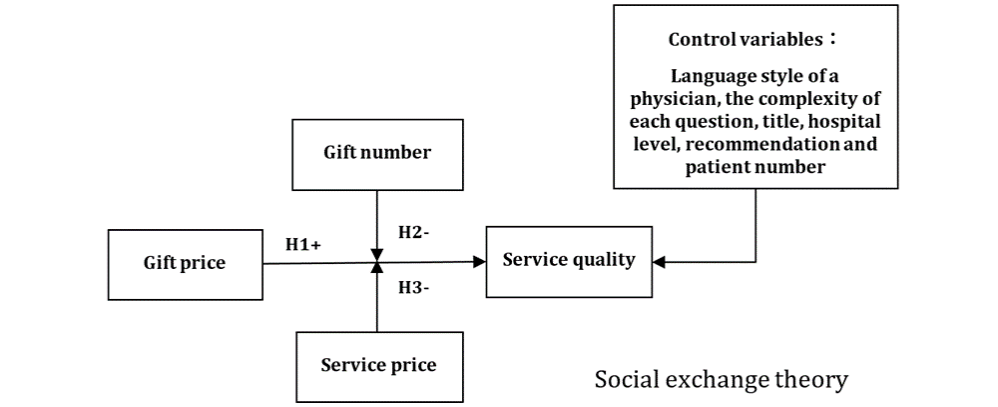
Research model and hypotheses based on social exchange theory. H1: hypothesis 1, gift price has a positive impact on the quality of physicians’ online consultation services; H2: hypothesis 2, the number of gifts that physicians receive negatively moderates the impact of gift price on the quality of physicians’ online consultation service; H3: hypothesis 3, service price negatively moderates the relationship between gift price and the quality of physicians’ online consultation services.

Based on the above hypotheses, we constructed the research model that is summarized in Figure 1.

## Methods

### Research Context

Founded in 2006, Good Physician Online (haodf.com) is the largest online consultation service platform in China. By December 2018, 580,000 physicians from 9379 regular hospitals were participating on the website [39]. Good Physician Online is also the most popular platform for online gift behaviors, and the number of gifts that physicians receive can reach up to several thousand. Hence, we selected this website as the data source for our research.

**Figure 2:**
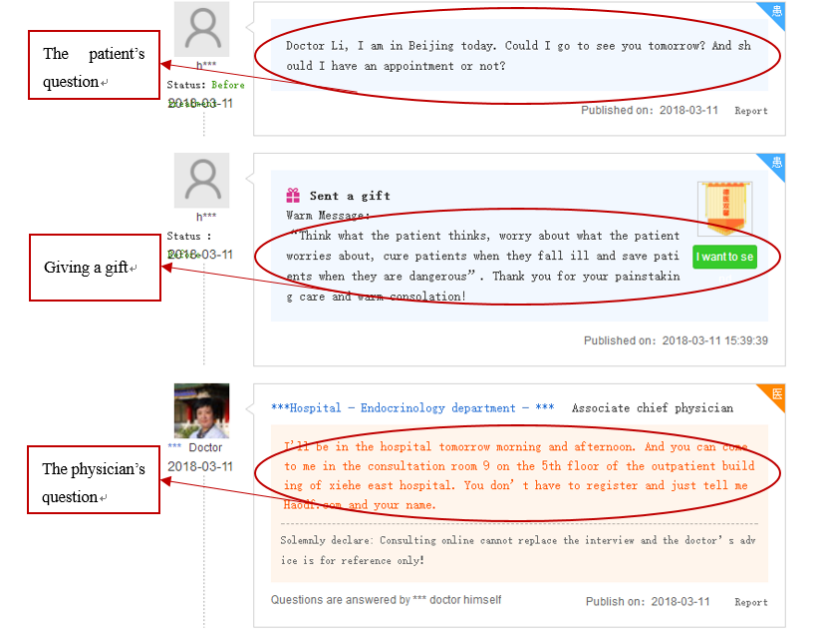
Examples of physician-patient interactions representing gift-giving behavior.

The main online consultation services provided by physicians in Good Physician Online include written consultation, telephone consultation, and appointment service. In a written consultation, patients need to first pay before they are able to communicate with physicians through text or pictures if necessary. In addition, patients can also purchase online gifts for physicians on the platform to express emotions. Representative examples of the physician-patient interaction and gift-giving behavior are shown in Figure 2.

### Data Collection

To eliminate the effects related to seeking consultations for different diseases, we collected data for physicians who only treat diabetes for the following reasons. First, diabetes is one of the most common chronic diseases. According to the 8th edition of the Global Diabetes Map released by the International Diabetes Federation, approximately 425 million adults worldwide were suffering from diabetes in 2017, including 114 million in China, ranking first in the world [40]. Second, diabetes, as a typical chronic noncommunicable disease, has the characteristics of a long onset, complicated etiology, and high difficulty to cure. Therefore, patients with diabetes are more likely to establish long-term and stable online interactions with physicians.

To empirically validate our research model, we developed a crawler targeting physicians who mainly treat patients with diabetes on the Good Physician Online website from October 12 to 13, 2018. The dataset included the personal information of the physicians and the physician-patient text dialogs during consultations. Finally, 141 physicians were included in the dataset, and the number of gifts that were exchanged in the consultations was 4249.

### Variables and Empirical Model

Medical service quality has long been a primary focus of scholars in the medical field, including in an online health context. However, a standardized scientific measure of medical service quality in online contexts has not yet been developed. Many studies have focused on the assessment of online health information quality and thus proposed some mature evaluation methods and indicators [41-43]. Based on these methods, some scholars have established the evaluation criteria of online medical consultation services from the perspectives of linguistic characteristics and information quality [44]. However, such evaluation criteria are generally used in assessments of expert consultation because medical information is considered to be professional. Therefore, such evaluation criteria were not considered suitable for the large sample data used in our study.

In the field of electronic commerce, longer reviews often include more product details, which can reduce the uncertainty of product quality [45]. Likewise, a physician’s answer is the main determinant of his or her service quality because online consultation services in online health communities are mainly carried out in the form of patients providing a question and physicians providing an answer. Furthermore, the average word count of a physician’s answer in a dialog represents each answer’s information content in the dialog. Therefore, we used the average word count to measure the quality of physicians’ online consultation services (Phy_Answer) as the dependent variable of our model.

The independent variable was the gift price (Gift_Price): Good Physician Online provides gifts with different prices, in the range of 1 to 200 yuan, for patients to choose from.

In addition, the following two moderating variables were included in the model: (1) service price (Service_Price), as patients need to pay for physicians’ online consultation services, and (2) gift number (Gift_Number), which represents the total number of online gifts that physicians received.

Finally, we introduced the following control variables. The first was physicians’ language styles (Phy_Style), which was based on the average word count of each physician answer to control for differences caused by physicians’ different language styles. The second control variable was the complexity of patient questions (Ques_comp), since the severity of diseases could affect the complexity of questions, which was also controlled based on the average word count of each patient question in a dialog. The title (Title) was also controlled for in the model. Physicians’ titles represent their professional ability and offline reputation, and there are three types physicians that participate in Good Physician Online: chief physicians, associate chief physicians, and attending physicians. Since most of the physicians using the website are chief physicians, we combined the other two categories and used one dummy variable to measure physicians’ titles (see Table 1). The hospital level (Hospital_Level) was further controlled, which is evaluated by government health departments, and physicians working at hospitals of different levels have access to different medical resources. In Good Physician Online, hospital levels are classified as level A, B or C, with A being the highest quality. However, most of the hospitals that physicians on the website belong to are level A hospitals; therefore, we combined the other two categories and used hospital level as a dummy variable (Table 1). Recommendation was another control variable, which ranges along a scale of 0-5 with 5 being the best, and represents the comprehensive popularity of a physician. Finally, patient number was considered as the number of patients that a physician is caring for, which represents the physician’s workload. All of the variables mentioned above are defined in Table 1. In addition, we used multiple linear regression to test the model in SPSS software (SPSS Inc, Chicago, IL, USA), and took the logarithmic value of the patient number, gift price, service price, and gift number to stabilize the variance. Our empirical models are summarized as follows:

(1) Phy_Answer=*α_ij_* + *α_1_* × *Gift_Price_ij_* + *α_2_* × *Gift_Number_ij_* + *α_3_* × *Service_Price_ij_* + *α_4_* × *Control_j_* + *ε_j_*

(2) Phy_Answer=*α_ij_* + *α_1_* × *Gift_Price_ij_* + *α_2_* × *Gift_Number_ij_* + *α_3_* × *Service_Price_ij_* + *α_4_* × *Gift_Number_ij_* × *Gift_Price_ij_* + *α_5_* × *Service_Price_ij_* × *Gift_Price_ij_* + *α_6_* × *Control_j_* +*ε_j_*

where *i* denotes a dialog and *j* denotes a physician. *α_ij_* are the coefficients to be estimated. Control*_j_* represents the control variables for physician *j*, and *ε_j_* is the standard error.

**Table 1:** Descriptions of model variables.

Variables and symbols		Variable types
**Control variables**		
	Phy_Style	Counting variable
	Ques_comp	Counting variable
	Title	Binary variable (Title is chief physician: 1, otherwise: 0)
	Hospital_Level	Binary variable (Hospital level is A: 1, otherwise: 0)
	Recommendation	Counting variable
	Patient_number	Counting variable
**Independent variables**		
	Gift_Price	Counting variable
	Service_Price	Counting variable
	Gift_Number	Counting variable
**Dependent variable**		
	Phy_Answer	Counting variable

## Results

### Descriptive Statistics and Correlations

Descriptive statistics and correlations for the key variables used in the analysis are presented in Multimedia Appendix 1. The results showed a correlation between gift price and the quality of physicians’ online consultation service. In addition, correlations between the independent variable and control variables were relatively weak, which helped to yield more stable results.

### Empirical Results

The empirical results of the model are summarized in Table 2, demonstrating support for all three of our hypotheses.

Model 1 shows the fitting degree of control variables and the dependent variable (adjusted *R^2^*=0.04, *F* Change=17.003, *P*<.001). In Model 2 (adjusted *R^2^*=0.051, *F* Change=14.907, *P*<.001), gift price had a significant positive impact on the quality of physicians’ online consultation services, which supports hypothesis 1. In model 3 (*R^2^*=0.059, *F* change=15.079, *P*<.001), we added the interaction term of gift number and gift price based on model 2, showing that gift number plays a negative role in regulating the relationship between gift price and the quality of physicians’ online consultation service, which supports hypothesis 2. Similarly, the results of model 4 (adjusted *R^2^*=0.063, *F* change=16.613, *P*<.001) showed that service price negatively moderates the relationship between gift price and the quality of physicians’ online consultation service, supporting hypothesis 3.

**Table 2:** Model test results.

Variables	Model 1		Model 2			Model 3			Model 4	
	β (SE)	*P* value		β (SE)	*P* value		β (SE)	*P* value	β (SE)	*P* value
Intercept	117.974(26.243)	<.001		85.143 (27.589)	.002		19.946 (30.823)	.52	–16.145 (33.024)	.63
Phy_Style	1.119 (0.172)	<.001		1.075 (0.175)	<.001		1.067 (0.174)	<.001	1.087 (0.174)	<.001
Ques_comp	0.257 (0.057)	<.001		0.250 (0.058)	<.001		0.250 (0.057)	<.001	0.252 (0.057)	<.001
Title	–3.732 (2.945)	.21		–4.325 (2.946)	.14		–4.789 (2.935)	.10	–4.435 (2.928)	.13
Hospital_Level	–3.558 (3.680)	.33		–2.167 (3.721)	.56		–2.118 (3.705)	.57	–3.096 (3.702)	.40
Recommendation	–15.597 (7.536)	.04		–14.559 (7.511)	.05		–15.472 (7.480)	.04	–13.651 (7.466)	.07
Patient_Number	–5.166 (1.877)	.006		3.496 (3.124)	.26		4.181 (3.114)	.18	4.732 (3.113)	.13
Gift_Price	N/A^a^	N/A		4.941 (1.523)	.001		31.286 (5.852)	<.001	42.321 (6.960)	<.001
Service_Price	N/A	N/A		–4.852 (1.812)	.007		–5.184 (1.805)	.004	18.870 (4.672)	<.001
Gift_Number	N/A	N/A		–5.282 (2.165)	.02		7.407 (3.472)	.03	–6.487 (2.162)	.003
Gift_Number*Gift_Price	N/A	N/A		N/A	N/A		–5.080 (1.090)	<.001	N/A	N/A
Service_Price*Gift_Price	N/A	N/A		N/A	N/A		N/A	N/A	–9.245 (1.680)	<.001

^a^N/A: not applicable; the variable was not included in the model.

### Robustness Check

The quality of the information support paragraphs, instead of the complete answer, was used to test the robustness of our model. Specifically, we used the average word count of a physician’s answer to measure the quality of the physician’s online consultation service, but ignored the content included in the answer. However, online consultation services provided by physicians generally include two parts: information support and emotional support [46], which are the main functions in online health communities [47]. Therefore, we divided every answer of physicians into these two parts by manual annotation and explored the impacts of gift-giving behaviors on these components separately.

First, an expert was invited to define information support and emotional support according to related knowledge and real data. Information support was defined as the provision and exchange of information related to medical technology and medical process, such as diseases (diagnosis, prescription, treatment, and notes during treatment), hospitalization, registration, and others. In other words, information support refers to physicians answering professional questions and providing information. Emotional support was defined as expressions that are clearly not related to medical technology from physicians, such as gratitude, sympathy, comfort, support, encouragement, respect, courtesy, and responsibility. Care, supervision, active greetings, and offering active solutions to problems from physicians are all examples of emotional support; other cases should be judged flexibly according to the context. We then selected two graduate students with professional backgrounds to annotate 18,392 physician answers manually based on these definitions. The consistency check showed that the kappa value of information support was 0.85, whereas that of emotional support was 0.78. Finally, inconsistent data were annotated again by the expert. Descriptive statistics and correlations for all variables are shown in Multimedia Appendix 2. These results reflect that physicians typically pay more attention to patients’ information needs (mean 38.870) than to their emotional needs (mean 3.595). Therefore, we decided to use the information support of physicians to check the robustness of our model, and empirical results are presented in Table 3.

We found a significant positive impact between gift price and the information support of physicians (Model 1: *R^2^*=0.025, *F* Change=11.086, *P*<.001; Model 2: *R^2^*=0.033, *F* Change=9.767, *P*<.001; Model 3: *R^2^=*0.035, *F* change=9.457, *P*<.001; Model 4: *R^2^*=0.038, *F* change=10.297, *P*<.001). Moreover, both gift number and service price negatively moderated the impact of gift price on physicians’ information support. Hence, these three results are consistent with the main model, indicating that our model is robust. We also found that gift price positively affected the emotional support of physicians, but there was no moderating effect of gift number and service price (Model 5: *R^2^*=0.071, *F* change=20.913, *P*<.001; Model 6: *R^2^*=0.071, *F* change=18.298, *P*<.001).

**Table 3:** Model robustness test.

Variables	Model 1^a^		Model 2^a^		Model 3^a^		Model 4^a^		Model 5^b^		Model 6^b^	
	β (SE)	*P* value	β (SE)	*P* value	β (SE)	*P* value	β (SE)	*P* value	β (SE)	*P* value	β (SE)	*P* value
Intercept	68.062 (22.366)	.002	46.429 (23.563)	.05	15.961 (26.411)	.55	–13.749 (28.299)	.63	18.724 (3.320)	<.001	16.429 (3.999)	<.001
Phy_Style	0.767 (0.146)	<.001	0.741 (0.150)	<.001	0.737 (0.149)	<.001	0.748 (0.149)	<.001	0.017 (0.021)	.42	0.017 (0.021)	.42
Ques_comp	0.207 (0.049)	<.001	0.205 (0.049)	<.001	0.205 (0.049)	<.001	0.207 (0.049)	<.001	0.021 (0.007)	.003	0.021 (0.007)	.003
Title	–1.896 (2.510)	.45	–2.243 (2.516)	.37	–2.46 (2.515)	.33	–2.309 (2.509)	.36	–0.098 (0.355)	.78	–0.101 (0.355)	.78
Hospital_Level	1.230 (3.136)	.70	2.390 (3.178)	.45	2.413 (3.174)	.45	1.838 (3.172)	.56	–3.344 (0.448)	<.001	–3.365 (0.448)	<.001
Recommendation	–7.621 (6.423)	.24	–6.587 (6.415)	.31	–7.014 (6.409)	.27	–6.048 (6.398)	.35	–2.377 (0.904)	.009	–2.356 (0.904)	.009
Patient_Number	–3.328 (1.599)	.04	2.857 (2.668)	.28	3.177 (2.668)	.23	3.591 (2.667)	.18	–0.923 (0.376)	.01	–0.895 (0.377)	.02
Gift_Price	N/A^c^	N/A	2.720 (1.301)	.04	15.032 (5.014)	.003	24.929 (5.964)	<.001	0.939 (0.183)	<.001	1.786 (0.843)	.03
Service_Price	N/A	N/A	–3.954 (1.547)	.01	–4.110 (1.547)	.008	10.139 (4.004)	.01	0.584 (0.218)	.007	1.122 (0.566)	.05
Gift_Number	N/A	N/A	–3.675 (1.849)	.05	2.254 (2.975)	.45	–4.391 (1.853)	.02	–0.322 (0.260)	.22	–0.349 (0.262)	.18
Gift_NumberGift_Price	N/A	N/A	N/A	N/A	–2.374 (0.934)	.01	N/A	N/A	N/A	N/A	N/A	N/A
Service_PriceGift_Price	N/A	N/A	N/A	N/A	N/A	N/A	–5.493 (1.440)	<.001	N/A	N/A	–0.210 (0.203)	.30

^a^Information support.

^b^Emotional support.

^c^N/A: not applicable; the variable was not included in the model.

## Discussion

### Principal Findings

Our empirical results supported all three of our hypotheses. First, in online medical consultations, gift price positively affects the quality of physicians’ online consultation service. This suggests that in response to patients’ mental and economic efforts, physicians will reciprocate with better online service, which contributes to establishing more stable physician-patient relationships. This result confirms the existence of the principles of reciprocity and equality in online physician-patient interactions. Furthermore, these principles are also widespread in other online communities. For example, researchers have verified that an increase in the number of reciprocity messages the actor broadcasts in online social networks increases the reciprocity reactions from his or her audience [21]. Similarly, some scholars have found that there is a consistent reciprocal mode between the information users publish and the answer they receive in an online gaming community [48].

Second, the number of online gifts physicians receive plays a negative role in regulating the impact of gift price on the quality of physicians’ online consultation service, proving that a marginal diminishing effect exists not only in the field of economics but also in people’s productivity, life, and social management. Some researchers have studied the relationship between individual income and happiness based on this theory, proving that higher income increases happiness in developing countries, whereas this effect is minimal in developed countries [11].

**Figure 3:**
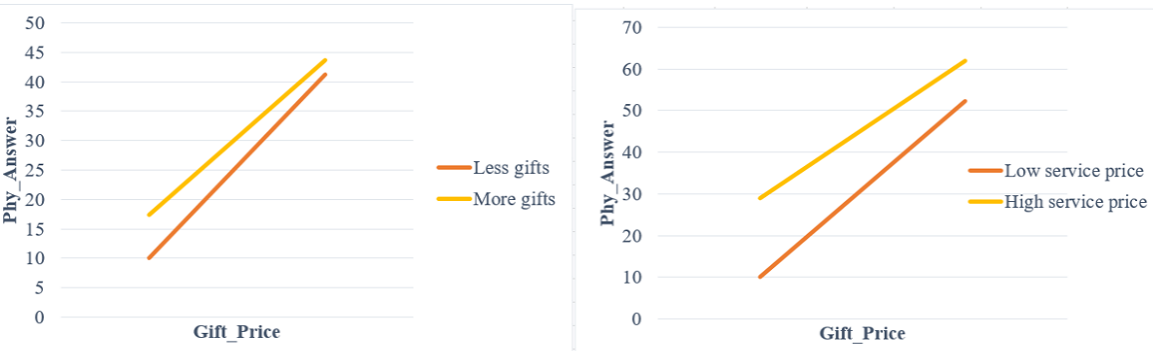
Interaction effects of gift number and service price.

Third, service price negatively moderates the relationship between gift price and the quality of physicians’ online consultation service. The gift price in Good Physician Online ranges from 1 to 200 yuan, accounting for about one quarter of the service price on average. In addition, the service price could be used as the comparison level for physicians when they judge the value of gifts because patients always pay for the consultation before sending gifts. Comparison level theory was originally developed to study people’s assessment of interpersonal relationships, such as the perception of marital relationships [49]. Subsequently, scholars have applied this theory in many different contexts. For example, researchers confirmed that workers’ reported satisfaction levels were inversely related to their comparison wage rates [50] and idealized advertising images reduced women’s satisfaction with their attractiveness [51]. Collectively, these studies demonstrated that comparison level theory is a psychological phenomenon that widely exists in several aspects of human society. For further explanation of these interaction effects, see Figure 3.

Finally, both information support and emotional support could be enhanced by physicians when they receive gifts with higher prices. However, compared with patients’ emotional needs, physicians in online health communities tend to pay more attention to their information needs.

### Theoretical Implications

Our study contributes new knowledge in several key ways. First, despite previous research about the impact of online gifts in social networks and live broadcast platforms [33,52-55], few scholars have paid attention to the gifts provided in online health communities. Furthermore, research related to gift-giving behaviors in an online health community verified that online gifts from patients could improve the speed of physicians’ answers during the consultation [8], but ignored the potential effect of gift price. Our study is among the first to use real data to empirically examine the effect of gift price in an online health community, which is a universally beneficial sector.

Second, SET was proposed in the 1960s and has since been widely applied in various fields. In recent years, SET has often been used to explain user behaviors in online contexts [22,56-58]. Our study further adds to this literature by verifying that the physician-patient interaction is also a form of social exchange.

Third, scholars have long highlighted the importance of emotional support [59], but few studies have empirically investigated this assumption. Our study revealed the actual situation of information and emotional support in the online medical field using a manual annotation approach. In addition, the text of physician-patient interactions contains an abundance of valuable information, which has inspired us to adopt a text mining analysis in future research.

### Practical Implications

The original intention of online gifts in online health communities is to provide patients with a channel to express gratitude to physicians. However, this channel has been questioned since its launch from an ethics perspective because physicians can receive economic benefits from this practice. Although patients purchase gifts voluntarily, they may still feel embarrassed and uncomfortable [5]. Moreover, some physicians have recommended refusing gifts from patients under the pressure of public opinion and professional ethics [60,61]. All of these questions motivated our reflections about the validity of online gifts in online health communities.

The results of our study indicate that gift price has a significant positive impact on the quality of physicians’ online consultation service, and the impact is stronger for physicians that receive fewer gifts or a lower service price. These conclusions could provide practical guidance for patients, but such speculation is beyond the scope of this study. Furthermore, we do not encourage sending gifts with higher prices to physicians as this may lead to unhealthy gift-giving trends in online health communities and undermine online physician-patient relationships. Thereby, we propose some suggestions from the perspective of platform management. First, compared with service price, the gift price in some online health communities is sufficiently high to affect the interactions between physicians and patients; thus, we recommended lowering the gift price or narrowing the price range to mitigate this impact. Second, we suggest that more value should be placed on the function of emotional expression. Specifically, patients could be granted more permissions such as sending images or voice messages when they write their greetings associated with gifts. Third, in addition to online gifts, other free channels for emotional expression could be provided to relieve the discomfort of patients.

Finally, the results of our robustness check revealed that physicians pay more attention to patients’ information needs rather to their emotional needs when they provide online consultation services. However, emotional support is more effective than information support in alleviating patients’ poor conditions in online health communities [62]. Therefore, we recommend that physicians could attach more importance to patients’ emotional needs during online consultations, which could contribute to achieving a better treatment effect and improved patient satisfaction.

### Limitations and Future Research

Several limitations and prospects of this study must be considered. First, we focused on only one context, which helps us improve the internal validity but may also reduce the generalizability of our findings. Therefore, future research should be performed to validate our results in other contexts. Second, there is no available mature criterion to measure online medical service quality. In this study, we used both the word count and content of physicians’ answers to measure service quality. However, it is still difficult to fully represent the actual quality of physicians’ services. In the future, we will continue to explore new ways to measure the quality of physicians’ online medical services. Third, the possible cumulative effect of gifts was ignored in our study, which could also be explored in future research. Fourth, multiple methods should be used to understand the true significance of these key findings, such as a quality study.

### Conclusions

Despite the original intention of online gifts as offering a new channel for patients to thank physicians, people remain suspicious of the validity of online gifts. Our study offers a better understanding of the impact of online gifts and contributes to settling existing disputes in this field. We found that the quality of physicians’ online consultation service could be affected by the price of gifts received from patients, which implies that online gifts are more than a simple channel for patients to express their emotions. However, we hold that it is important to consider online gifts from an objective and rational perspective since online gifts in online health communities are still new. We believe that this paper can help provoke new ideas and perspectives about the validity of online gifts in online health communities.

## Appendix

Multimedia Appendix 1 Descriptive statistics and correlations.

Multimedia Appendix 2 Descriptive statistics and correlations in robustness check.

## Abbreviations

SET: social exchange theory
