# Supplementary material for: Impact of the Price of Gifts From Patients on Physicians’ Service Quality in Online Consultations: Empirical Study Based on Social Exchange Theory
Source: J Med Internet Res. 2020 May 5;22(5):e15685. doi: 10.2196/15685 (PMC7238091; doi:10.2196/15685)
Supplement: Multimedia Appendix 1 [file jmir_v22i5e15685_app1.docx]

| Variable | Min | Max | Mean | S.D. | 1 | 2 | 3 | 4 | 5 | 6 | 7 | 8 | 9 |  |
| --- | --- | --- | --- | --- | --- | --- | --- | --- | --- | --- | --- | --- | --- | --- |
| 1. Phy_Style | | 5.667 | 54.955 | 22.123 | 0.149 |  |  |  |  |  |  |  |  |  |
| 2. Ques_comp | | 2.000 | 479.000 | 32.503 | 0.415 | 0.029 |  |  |  |  |  |  |  |  |
| 3. Title | | 0.000 | 1.000 | 0.701 | 0.458 | -0.177*** | 0.036* |  |  |  |  |  |  |  |
| 4. Hospital_Level | | 0.000 | 1.000 | 0.881 | 0.324 | 0.170*** | 0.099*** | -0.040** |  |  |  |  |  |  |
| 5. Recommendation | | 3.900 | 5.000 | 4.401 | 0.003 | 0.136*** | 0.069*** | 0.196*** | 0.196*** |  |  |  |  |  |
| 6. Patient_number | | 67.000 | 9251.000 | 2633.381 | 29.413 | -0.036 | 0.062*** | 0.355*** | 0.159*** | 0.639*** |  |  |  |  |
| 7. Gift_price | | 0.000 | 200.000 | 22.361 | 0.486 | 0.015 | 0.064*** | 0.053*** | 0.028 | 0.049** | -0.013 |  |  |  |
| 8. Service_price | | 0.000 | 659.000 | 82.245 | 1.325 | 0.093*** | 0.119*** | 0.137*** | 0.208*** | 0.377*** | 0.367*** | 0.093*** |  |  |
| 9. Gift_Number | | 2.000 | 1225.000 | 473.862 | 6.253 | -0.101*** | -0.011 | 0.327*** | 0.121*** | 0.521*** | 0.839*** | 0.019 | 0.455*** |  |
| 10. Phy_Answer | | 0.000 | 1135.000 | 46.947 | 1.401 | 0.138*** | 0.063*** | 0.016 | 0.033* | -0.029 | -0.033* | 0.113*** | -0.011 | 0.007 |

*p<0.05, **p<0.01, ***p<0.001.
